# Antioxidant and Anticancer Aporphine Alkaloids from the Leaves of *Nelumbo nucifera Gaertn.* cv. *Rosa-plena*

**DOI:** 10.3390/molecules191117829

**Published:** 2014-11-03

**Authors:** Chi-Ming Liu, Chiu-Li Kao, Hui-Ming Wu, Wei-Jen Li, Cheng-Tsung Huang, Hsing-Tan Li, Chung-Yi Chen

**Affiliations:** 1 Tzu Hui Institute of Technology, Pingtung County 92641, Taiwan; E-Mails: beagleliu@gmail.com (C.-M.L.); joe7day@yahoo.com.tw (C.-L.K.); 2 School of Medical and Health Sciences, Fooyin University, Ta-Liao District, Kaohsiung 83102, Taiwan; E-Mails: mt019@fy.edu.tw (H.-M.W.); mt082@fy.edu.tw (W.-J.L.); 3 St. Joseph Hospital Dental Department, Kaohsiung 802, Taiwan

**Keywords:** *Nelumbo nucifera* Gaertn. cv. *Rosa-plena*, aporphine, antioxidation, 7-hydroxydehydronuciferine

## Abstract

Fifteen compounds were extracted and purified from the leaves of *Nelumbo nucifera Gaertn.* cv. *Rosa-plena*. These compounds include liriodenine (**1**), lysicamine (**2**), (−)-anonaine (**3**), (−)-asimilobine (**4**), (−)-caaverine (**5**), (−)-*N*-methylasimilobine (**6**), (−)-nuciferine (**7**), (−)-nornuciferine (**8**), (−)-roemerine (**9**), 7-hydroxydehydronuciferine (**10**) cepharadione B (**11**), β-sitostenone **(12**), stigmasta-4,22-dien-3-one **(13**) and two chlorophylls: pheophytin-a **(14**) and aristophyll-C **(15**). The anti-oxidation activity of the compounds was examined by antiradical scavenging, metal chelating and ferric reducing power assays. The results have shown that these compounds have antioxidative activity. The study has also examined the antiproliferation activity of the isolated compounds against human melanoma, prostate and gastric cancer cells. The results shown that 7-hydroxydehydronuciferine (**10**) significantly inhibited the proliferation of melanoma, prostate and gastric cancer cells. Together, these findings suggest that leaves of *Nelumbo nucifera Gaertn.* cv*. Rosa-plena* are a good resource for obtaining the biologically active substances with antioxidant properties.

## 1. Introduction

*Nelumbo nucifera Gaertn*, called lotus, is a perennial aquatic crop grown and widely distributed in Asia and some countries in Africa [1]. The leaves, fruits, seeds, stamens, and roots of lotus are used as a food garnish and traditional medicine in China. In China, lotus leaves are used to treat hematemesis, hematuria, hyperlipidemia and obesity. Previous studies have shown that aporphine alkaloids from the leaves of *Nelumbo nucifera Gaertn* have many pharmacological properties such as anti-diabetic, anti-obesity, anti-hyperlipidemic, anti-oxidant, and anti-HIV activities [2,3,4,5,6,7]. *Nelumbo nucifera Gaertn.* cv*. Rosa-plena* is very similar to *Nelumbo nucifera Gaertn*. We hypothesized that *Nelumbo nucifera Gaertn.* cv*. Rosa-plena* would also contain abundant aporphine alkaloids.

Free radicals are the main factor of oxidative damage in animals and humans. Reactive oxygen species (ROS) are formed by hydrogen peroxide or superoxide anions. The increased production of ROS can result in oxidative stress. The excess of ROS can cause DNA damage and be harmful to cells and tissues [8]. A lot of studies have reported that many diseases are associated with ROS production, such as coronary heart disease and cancers [9,10,11]. A dietary supplement intake of antioxidants such as vitamins A, C and E can prevent ROS production. Plants possess active ingredients with many non-vitamin antioxidants that can also prevent ROS production [12]. Therefore, more and more attention has been focused on natural products.

**Figure 1:**
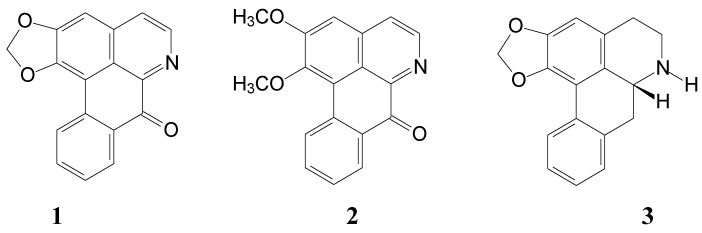

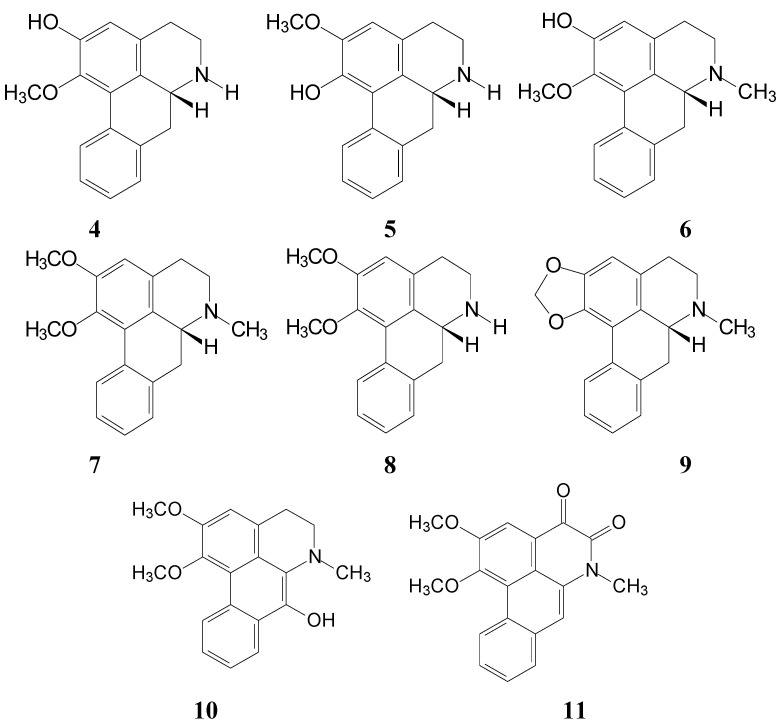
Structures of alkaloids 1–11 isolated from the leaves of *N. nucifera*.

In a previous study we have shown that aporphine derivatives in a leave extract of *N. nucifera* Gaertn. cv. *Rosa-plena* have anthelmintic activities against *Anisakis simplex* and *Hymenolepis nana* [13]*.* In the present study, we describe the isolation and characterization of several compounds from the leaves of *N. nucifera* Gaertn. cv. *Rosa-plena.* These compounds are aporphine alkaloids: liriodenine (**1**) [14], lysicamine (**2**) [14], (−)-anonaine (**3**) [14], (−)-asimilobine (**4**) [15], (−)-caaverine (**5**) [16], (−)-*N*-methylasimilobine (**6**) [15], (−)-nuciferine (**7**) [17], (−)-nornuciferine (**8**) [14], (−)-roemerine (**9**) [17], 7-hydroxydehydronuciferine (**10**) [18] and cepharadione B (**11**) [19]; two steroids: β-sitostenone (**12**) [15] and stigmasta-4,22-dien-3-one (**13**) [15]; and two chlorophylls: pheophytin-a (**14**) [20] and aristophyll-C (**15**) [21]. The structures of the alkaloids are summarized in Figure 1.

We have found no publications concerning the anti-oxidant activity of the chemical components of this plant or their activity against cancer cell growth, so the objective of the present study was thus to investigate the anti-oxidative and anti-cancer activity of these compounds.

## 2. Results and Discussion

### 2.1. Antioxidant Activities of Compounds from N. nucifera Gaertn. cv. Rosa-plena

Radical scavenging was measured in this study as antioxidants act to inhibit oxidation. DPPH is a stable free radical used to evaluated the radical scavenging activity of compound. The mechanism of its radical scavenging activity is the antioxidant transfer of an electron or a hydrogen atom to DPPH. The ABTS assay evaluates the quenching and anti-oxidation activity of compounds depending on hydroxyl group substitution and number of aromatic rings. As shown in Table 1, (−)-*N*-methylasimilobine (**6**) and lysicamine (**2**) have radical scavenging activity comparable with vitamin C at the same dose (100 μM) in the ABTS and DPPH assays. Iron is essential for oxygen transport and respiration enzyme activity in the human body. However, iron induces oxidative damage in tissues and cells. The reducing power assay is used as a reflection of antioxidant activity. The ferric reducing power antioxidant assay is an assay to measure the reducing potential of an antioxidant reacting with a ferric 2,4,6-tripyridyl-S-triazine (Fe(III)-TPTZ) complex, which produces a dark blue colored ferrous Fe(II)-TPTZ complex. In the presence of chelating agents, the reagent complex formation is disrupted, resulting in a reduction of its color. The ferrous ion chelating activities of the tested compounds at 100 μM compared with 3-*tert*-butyl-4-hydroxyanisole (BHA) as the reference compound are also listed in Table 1. Only (−)-nuciferine (**7**) and pheophytin-A (**14**) displayed minor effects in the chelating assay. Lysicamine (**2**) displayed modest ferric reducing power. In addition, the radical scavenging activity of the MeOH extract of leaves is 35.6% and 40.2% by the ABTS and DPPH assays. The ferrous ion chelating activities of the MeOH extract of leaves is 13.3%. These results suggest the radical scavenging activity of MeOH extract of leaves is mainly a result of the presence of (−)-*N*-methylasimilobine (**6**) and lysicamine (**2**).

**Table 1:** Antioxidant activity of the extracted compounds at 100 μM.

Compounds	ABTS (%)	DPPH (%)	Chelating (%)	Reducing Power (OD 700)
Liriodenine (**1**)	12.9	na	3.4	0.105
Lysicamine (**2**)	6.4	24.7	7.8	0.302
(−)-Anonaine (**3**)	14.2	na	1.7	0.128
(−)-Asimilobine (**4**)	12.4	3.9	5.2	0.109
(−)-Caaverine (**5**)	na	na	7.8	0.225
(−)-*N*-Methylasimilobine (**6**)	23.5	na	6.9	0.116
(−)-Nuciferine (**7**)	2.8	9.1	11.3	0.110
(−)-Nornuciferine (**8**)	5.6	16.9	7.8	0.075
(−)-Roemerine (**9**)	1.8	na	1.7	0.090
7-Hydroxydehydronuciferine (**10**)	5.3	na	na	0.120
Cepharadione B (**11**)	3.2	5.0	7.5	0.135
β-Sitostenone (**12**)	na	1.3	na	-
Phenophytin-A (**14**)	6.8	1.2	10.4	-
Aristophyll-C (**15**)	na	1.3	6.9	-
MeOH extract	35.6	40.2	13.3	-
Vitamin C	97	100	-	-
EDTA	-	-	26.8	-
BHA	-	-	-	0.603

Data in the table are means ± S.D. of *n* = 3; -: no testing; na: not active.

### 2.2. Anti-Proliferation of Compounds in Cancer Cells and Human Dermal Fibroblast Cells

**Figure 2:**
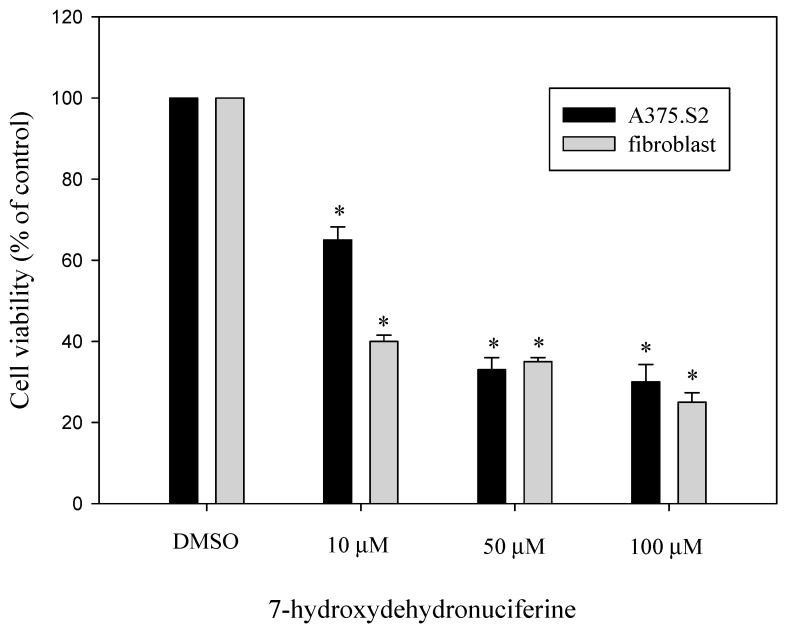
Anti-proliferative effects of compounds on A375.S2 and human dermal fibroblasts cells. Cell growth was determined by a MTT assay after incubation with 10, 50, 100 μM of 7-hydroxydehydronuciferine (**10**), respectively. Results are expressed as the percent of the cell proliferation of the vehicle control at 24 h. Values are expressed as means ± S.D. of *n* = 3. * *p* < 0.05 *versus* DMSO group.

The cytoxicity of compounds was determined by a standard MTT assay. AGS and DU-145 are human gastric and prostate cancer cell lines, respectively. The results show that only 7-hydroxy-dehydronuciferine (**10**) has significant cytoxicity in AGS and DU-145 cells (Table 2), with IC_50_ values of 62.9 ± 0.1 μM and 80.8 ± 0.2 μM, respectively. The melanoma cell line A375.S2 cell line is metastatic and widely used in anti-melanoma studies. We further examined the cytoxicity of 7-hydroxydehydronuciferine (**10**) against A375.S2 cells and human dermal fibroblast cells after a 24 h treatment (Figure 2). 7-Hydroxydehydronuciferine (**10**) had significant cytotoxicity in the A375.S2 cell line, but it also has cytotoxic activity in normal cells (Figure 2).

**Table 2:** Anti-proliferative effects of aporphine alkaloids on AGS and DU-145 cells.

Sample	IC_50_ (µM)	
	AGS	DU-145
Liriodenine (**1**)	˃500	95.4 ± 0.4
Lysicamine (**2**)	˃500	˃500
(−)-Anonaine (**3**)	˃500	150.1 ± 0.3
(−)-Asimilobine (**4**)	˃500	˃500
(−)-Caaverine (**5**)	˃500	94.4 ± 0.1
(−)- *N*-Methylasimilobine (**6**)	˃500	˃500
(−)-Nuciferine (**7**)	˃500	218.4 ± 0.5
(−)-Nornuciferine (**8**)	˃500	˃500
(−)-Roemerine (**9**)	˃500	˃500
7-Hydroxydehydronuciferine (**10**)	62.9 ± 0.1	80.8 ± 0.2
Cepharadione B (**11**)	˃500	na

Data in the table are means ± S.D. of *n* = 3; na: no testing.

## 3. Experimental Section

### 3.1. General Procedures

UV spectra were obtained on a Jasco UV-240 spectrophotometer (Jasco, Tokyo, Japan) in MeCN. IR spectra were measured on a Hitachi 260-300 spectrophotometer (Hitachi, Tokyo, Japan). ^1^H-NMR (400/500 MHz) and ^13^C-NMR (100 MHz), HSQC, HMBC, COSY and NOESY spectra were obtained on a Varian Unity Plus NMR spectrometer (Varian, San Francisco, CA, USA). For each sample, 128 scans were recorded with the following settings: 0.187 Hz/point; spectra width, 14400 Hz; pulse width, 4.0 μs; relaxation delay, 2 s. Low-resolution ESI-MS spectra were obtained on an API 3000 (Applied Biosystems, Foster City, CA, USA) and high-resolution ESI-MS spectra on a Bruker Daltonics APEX II 30e spectrometer (Bruker, Bremen, Germany). Silica gel 60 (Merck, 70–230 mesh, 230–400 mesh, Darmstad, Germany) was used for column chromatography. Precoated silica gel plates (Merck, Kieselgel 60 F-254), 0.20 mm and 0.50 mm, were used for analytical TLC and preparative TLC, respectively, and visualized with 10% H_2_SO_4_.

### 3.2. Plant Material

The leaves of *Nelumbo nucifera* Gaertn. cv. *Rosa-plena* were collected from Tainan, Taiwan, in November 2008. Plant material was identified by Dr. Fu-Yuan Lu (Department of Forestry and Natural Resources College of Agriculture, National Chiayi University, Chiayi, Taiwan). A voucher specimen (*N. nucifera* Gaertn. cv. *Rosa-plena*) was deposited in the School of Medical and Health Sciences, Fooyin University, Kaohsiung, Taiwan.

### 3.3. Extraction and Isolation

The air-dried leaves of *N. nucifera* Gaertn. cv. *Rosa-plena* (1.5 kg) were extracted with MeOH (50 L × 5) at room temperature for three days and a MeOH extract (108.7 g) was obtained upon concentration under reduced pressure. The MeOH extract, suspended in H_2_O (1 L), was partitioned with CHCl_3_ (3 L × 4) to give fractions soluble in CHCl_3_ (57.2 g) and H_2_O (43.6 g). The CHCl_3_-soluble fraction was chromatographed over silica gel (1700 g, 70–230 mesh) using *n*-hexane/EtOAc/MeOH mixtures as eluents to produce six fractions. Part of fraction 3 (7.83 g) was subjected to silica gel chromatography, by eluting with *n*-hexane-acetone (7:1), enriched gradually with acetone, to furnish two fractions (3-1–3-2). Fraction 3-2 (2.13 g) was further purified on a silica gel column using *n*-hexane/acetone mixtures to obtain (−)-caaverine (**5**, 4 mg). Part of fraction 4 (10.27 g) was subjected to silica gel chromatography by eluting with *n*-hexane-acetone (5:1), enriched with acetone to furnish three further fractions (4-1–4-3). Fraction 4-1 (4.37 g) was further purified on a silica gel column using *n*-hexane-acetone mixtures to obtain lysicamine (**2**, 15 mg), 7-hydroxydehydronuciferine (**10**, 12 mg) and (−)-nornuciferine (**8**, 17 mg). Fraction 4-2 (3.05 g) was further purified on a silica gel column using *n*-hexane-acetone mixtures to obtain (−)-roemerine (**9**, 6 mg), (−)-nuciferine (**7**, 20 mg), (−)-anonaine (**3**, 5 mg) and cepharadione B (**11**, 15 mg). Fraction 4-3 (2.51 g) was further purified on a silica gel column using *n*-hexane-acetone mixtures to obtain (−)-asimilobine (**4**, 4 mg) and (−)-*N*-methylasimilobine (**6**, 16 mg). Part of fraction 5 (5.34 g) was subjected to silica gel chromatography by eluting with CH_2_Cl_2_/MeOH (40:1), enriched with MeOH to furnish two fractions (5-1–5-2). Fraction 5-1 (2.53 g) eluted with CH_2_Cl_2_/MeOH (30:1), was further separated using silica gel column chromatography and preparative TLC [CH_2_Cl_2_/MeOH (40:1)] and gave liriodenine (**1**, 4 mg).

### 3.4. Determination of DPPH Radical Scavenging Capacity

DPPH is a stable free radial with a violet color (absorbance at 517 nm) that changes to light yellow when the free radicals are scavenged. Various concentrations of the four compounds were added to 0.1 mL of stable DPPH (60 μM) solution. When DPPH reacts with hydrogen-donating antioxidant, it is reduced, resulting in a decrease in absorbance at 517 nm. The analyzed time interval was 10 min per point, up to 30 min by using UV-Vis spectrophotometer (Jasco). Vitamin C was used as a positive control. The DPPH• radical scavenging activity (%) was determined as: 1 − [(*A*_control_ − *A*_sample_)/*A*_control_] × 100.

### 3.5. ABTS^+^ Radical Scavenging Assay

The radical-scavenging activity of tested compounds against ABTS^+^ was assayed according to methods described previously [22]. Briefly, ABTS^+^ was dissolved in deionized water to 7 mM in concentration (pH = 7.4) and was then mixed with 2.45 mM potassium persulfate. The scavenging activity was determined by mixing with 180 μL of ABTS^+^and 40 μL of testing samples, and followed by measuring at absorbance 734 nm at 10 min. Ascorbic acid was used as a standard. The ABTS^+^ radical scavenging activity (%) was determined as: 1 − [(*A*_control_ − *A*_sample_)/*A*_control_] × 100.

### 3.6. Metal Chelating Activity

The ferrous ion chelating potential of the four *L. tulipifera* compounds was investigated according to a previously described method [9]. Briefly, various test concentrations of samples dissolved in DMSO were added to a solution of 2 mM FeCl_2_•4H_2_O (0.01 mL). The reaction was initiated by the addition of 5 mM ferrozine (0.02 mL), and the mixture was vigorously shaken and left standing at room temperature for 10 min. The absorbance of the mixture was then read at 562 nm against a blank. EDTA was used as a positive control. The metal chelating activity was determined as: 1 − [(*A*_control_ − *A*_sample_)/*A*_control_] × 100.

### 3.7. Reducing Power

The reducing powers of our natural pure compounds were determined according to the method of [9]. Briefly, various concentrations of test samples were mixed with 67 mM phosphate buffer (pH 6.8, 0.085 mL) and 20% potassium ferricyanide [K_3_Fe(CN)_6_, 2.5 μL) The mixture was incubated at 50 °C for 20 min, and trichloroacetic acid (10%, 0.16 mL) was then added to the mixture that was then centrifuged for 10 min at 3000 *g*. The upper layer of the solution (75 μL) was mixed with 2% FeCl_3_ (25 μL), and the absorbance was measured with a 96-well plate spectrophotometer at 700 nm. A higher absorbance demonstrates a higher reductive capability.

### 3.8. Cell Culture

Human melanoma cell lines A375.S2 and human dermal fibroblasts cell were obtained from the American Type Cell Culture Collection (ATCC, Manassas, VA, USA). DU-145 and AGS were obtained from BCRC (Bioresoure Collection and Research Center, Hsinchu, Taiwan). A375.S2 was cultured in DMEM supplemented with 10% FBS, 10 µg/mL of penicillin, 10 µg/mL of streptomycin and 0.25 μg/mL of amphotericin B. Human dermal fibroblasts cells were cultured in Fibroblast Growth Kit-Low Serum (ATCC^®^ PCS-201-041). DU-145 was culture in Minimum essential medium Eagle with 10% FBS. AGS was cultured in Ham’s F-12K medium with 10% 10% FBS. The cells were maintained in a humidified incubator at 37 °C in 5% CO_2_.

### 3.9. Cell Viability Assay—MTT Assay

The MTT assay was used to determine cell viability and proliferation. The cell lines were seeded in 96-well culture plates (1 × 104 cells/well). After seeding cells for 24 h, various compounds with different concentrations were added. After the treatment, the medium was replaced with fresh medium without drugs. MTT solution (5 mg/mL and dissolved in phosphate buffered saline; PBS) was diluted 1:10 in culture medium and added to a culture dish followed by an incubation at 37 °C. After 2 h of MTT treatment, the media was removed and each precipitate in a specific dish was dissolved in 100 μL of DMSO to dissolve the purple formazan crystals. After the dishes were gently shaken for 20 min in the dark to ensure maximal dissolution of formazan crystals, the optical density (OD) values of the supernatant were measured at 595 nm. All experiments were repeated at least three times. In consideration of the possible anti-proliferative effects of DMSO, a maximal amount (0.5%) of DMSO was added to culture and used as positive controls. DMSO at this amount was found not to affect the growth of cells.

### 3.10. Statistical Analysis

All experiments were carried out at three times and at least triplicate. The results were expressed as the average of the mean values ± standard deviation (SD), and statistical comparisons were carried out using one-way analysis of variance (ANOVA). A *p*-value < 0.05 was considered to indicate statistical significance. Analysis of the data and plotting of the figures were done with SigmaPlot software (Version 8.0, SPSS Scientific, Chicago, IL, USA) and SigmaStat (Version 2.03, SPSS Scientific) run on an IBM-compatible computer.

## 4. Conclusions

Free radicals are highly reactive chemicals and harmful to cells. At high concentrations free radicals cause cell damage, including DNA, proteins, and cell membranes damage. The damage to cells caused by free radicals may play a role in the development of cancer and other diseases. Antioxidants are known as free radical scavengers. Antioxidants neutralize free radicals, thus preventing them from causing damage. In this study, we isolated many aporphine alkaloids with antioxidant activity from the leaves of *Nelumbo nucifera Gaertn*. The antioxidant activity of these compounds was examined by DPPH, ABTS, chelating and reducing power assays*.* The overall results of the present study indicate that (−)-*N*-methylasimilobine (**6**), lysicamine (**2**), (−)-nuciferine (**7**) and pheophytin-a (**14**) have moderate antioxidant values in the DPPH, ABTS, chelating, and/or reducing power assays. Further, we examined the anti-proliferation activity of these compounds in cancer cells. It is interesting that 7-hydroxydehydronuciferine (**10**) has potent anti-cancer activity against prostate, gastric and skin cancer cell lines, but it possesses minor antioxidant activity. Apoptosis is a form of programmed cell death. Apoptosis occurs when the cells are exposed to some agents, so it is considered as a strategy to inhibit the proliferation of cancer cells. Until now, there are no publications about the activity of 7-hydroxydehydronuciferine (**10**) against cancer cells, and further studies of the mechanism(s) of action of 7-hydroxydehydronuciferine (**10**) against cancer growth should be examined in the future.
